# Evaluation of various obturation techniques with bioceramic sealers in 3D-printed C-shaped canals

**DOI:** 10.1186/s12903-024-04334-2

**Published:** 2024-05-12

**Authors:** Maryam Gharechahi, Melika Hoseinzadeh, Saeed Moradi, Mina Mehrjouei

**Affiliations:** 1 Department of Endodontics, Faculty of Dentistry, Dental Materials Research Center, Mashhad University of Medical Sciences, Mashhad, Iran; 2 Dentist, Research Assistant, Dental Research Center, Mashhad Dental School, Mashhad University of Medical Sciences, Mashhad, Iran; 3 Department of Endodontics, School of Dentistry, Iran University of Medical Sciences, Tehran, Iran

**Keywords:** Sealer, Ultrasonic, Calcium silicate, Mineral trioxide aggregate

## Abstract

This in vitro study compared various obturation techniques with bioceramic sealers for filling C-shaped 3D-printed replicas. A mandibular molar with a C-shaped root canal with a C1 configuration was obtained. After instrumenting with M3 Pro Gold files (United Dental, Shanghai, China) up to size #30/0.04, a CBCT scan of the tooth was taken. Sixty 3D-printed replicas of the tooth were created. The samples were obturated with EndoSeal TCS sealer (E. TCS; Maruchi, Wonju, Korea) or EndoSeal MTA (E. MTA; Maruchi, Wonju, Korea) (*n* = 30). The samples in each group were obturated with the following techniques (*n* = 10): (1) single-cone technique (SC), (2) SC with ultrasonic activation (UA), and (3) cold hydraulic compaction (CHC). Following incubation, the replicas’ apical, middle, and coronal thirds were inspected under a digital microscope, and the proportion of filling material and void were calculated. Also, the obturation time and sealer extrusion were recorded. Data were analyzed using ANOVA, LSD post-hoc, and the chi-square tests (α = 0.05). The results indicated that in the apical third, E. TCS-SC, E. TCS-UA, and E. MTA-UA had the lowest void percentage among groups (*p* < 0.05). In the middle thirds, samples obturated with E. TCS-UA showed a significantly lower void percentage among all groups (*p* < 0.05). However, in the coronal third, E. TCS-CHC showed the least void percentage (*p* < 0.05), followed by E. TCS-UA and E. MTA-CHC. The E. TCS-SC and E. TCS-UA were the least time-consuming methods (*p* < 0.05). Sealer extrusion significantly differed among the groups, with E. MTA-UA and E. TCS-UA showing higher incidence (*p* = 0.019). It was concluded that E. TCS-UA was the most convenient obturation technique. However, care must be taken when obturating the canals with high flow and ultrasonic activation near the vital anatomical landmarks.

## Introduction

The prevalence of C-shaped canals in mandibular first and second molar teeth is reported to be 0.3% and 12%, respectively [1]. Clinicians often face challenges during root canal treatment of these teeth due to the complex root canal system that changes during its course to the apex [2]. In these cases, shaping, disinfection, and root canal filling can be quite complicated and can, if not carried out perfectly, result in root canal microleakage, which is reported to be the leading cause of treatment failure in C-shaped canals [3]. In this instance the nature of the endodontic sealer, as well as, the obturation technique used, could influence the adaptation of the filling material to the C-shaped root canal walls and prevent bacterial penetration into the root canal system [3].

Although there are different classes of root canal sealers, recently, bioceramic sealers have gained popularity in modern root canal treatments due to their biochemical and biological properties and ease of use [4]. They are biocompatible and bond chemically to dentin due to high amounts of calcium and phosphate and create a crystal structure similar to hydroxyapatite [5, 6]. Endoseal MTA sealer (E. MTA; Maruchi, Wonju, Korea) is an MTA-based material, and its biocompatibility, antibacterial properties, and sealing ability have been reported previously [7]. EndoSeal TSC (E. TCS; Maruchi, Wonju, Korea) is one of the most recent sealers based on tricalcium silicate, which has good biocompatibility and radiopacity [8]. However, given these sealers’ differing flow and physicochemical properties, a detailed examination of various obturation techniques, including their ultrasonic activation in C-shaped canals, is warranted, although the literature is not unanimous in this regard [9, 10]. The uniform dispersion of the sealer, augmented bond strength to root dentin, and improved sealer penetration into the isthmus could be some of the benefits realized through ultrasonic device tips [11, 12]. The generated heat could improve the mixture of the sealer particles, increasing the cohesive strength between the particles [13], but could affect the chemical and physical features of these sealers [14]. Consequently, a consensus on the effectiveness of this activation technique remains elusive, attributed partly to variations in the chemical composition, viscosity, and flow of endodontic sealers, as well as differences in root canal anatomy [15, 16].

Cold hydraulic compaction (CHC), characterized by its straightforwardness and predictability, uses accessory gutta-percha cones as a plunger to disseminate the sealer into canal irregularities, thereby facilitating a more effective seal. Wisawawatin et al. [17] have found that obturating C-shape canals with calcium silicate-based sealers and the CHC method caused comparable gap volume compared to obturation with epoxy resin sealers and lateral or warm vertical compaction. However, there is a knowledge gap concerning the efficacy of the CHC technique compared with previous sealer activation. Addressing this, the present in vitro study was conducted to compare void and sealer percentages in three-dimensional (3D) printed C-shaped replicas with C1 configuration, exploring the performance of both E. MTA and E. TCS in conjunction with different obturation techniques.

## Methods and materials

The protocol of the present study was approved by the Research and Ethics Committee of Mashhad University of Medical Sciences (IR.MUMS.DENTISTRY.REC.1401.079).

### Sample selection

Fifteen extracted mandibular molars were obtained with fused roots. In order to select teeth with C1 anatomy according to the classification of Fan et al. [2], a CBCT scan was prepared using Planmeca ProMax® 3D Classic (PLANMECA OY, Helsinki, Finland). The scans were explored in the axial sections with a 0.125 mm thickness and 0.125 dimensions pixels in MicroDicom software. Eventually, a tooth with a C1 configuration throughout the canal was selected.

### Root canal preparation

After preparing the access cavity, three K-files #10 (Mani Inc., Japan) were inserted at the canal’s mesial, distal, and middle to measure the working length. The tooth was decoronated, and a 10 mm root length remained. The canal was instrumented by M3 Pro Gold rotary systems (United Dental, Shanghai, China) up to the size of #30/0.04 using an endomotor (NSK, Tokyo, Japan) based on the manufacturer’s instruction at 350 rpm and 1-Ncm torque. A K-file (MANI Inc., Tochigi, Japan) size #15 with a passive pressure was introduced to the canal to ensure root canal patency.

The canal isthmus was prepared using the anti-curvature method described by Abou-Rass et al. [18]. H-files (MANI Inc., Tochigi, Japan) sizes #15–25 were introduced to the canal and removed by pressuring the buccal wall to prevent strip perforation. After each file, 2 ml NaOCl 1% (Nik darman, Mashhad, Iran) was used. Finally, the root canal was irrigated by 10 ml EDTA 17% (Nik darman, Mashhad, Iran) and 2 ml saline (Samen, Mashhad, Iran). The canal was dried with paper points (Diadent, Cheongju-si, Korea).

### CBCT scanning and 3D Printing of the C-shaped Mandibular Molar

An instrumented sample was scanned using Planmeca ProMax® 3D Classic (PLANMECA OY, Helsinki, Finland) CBCT system at the 75-micron-accuracy mode. The tooth structure was reconstructed by Planmeca Romexis 6.2 software and saved in .stl format. The 3D model of the sample was transferred to an AccuFab-L4K 3D printer (SHINING 3D Technology Inc., Hangzhou, China). Sixty resin replicas with 4 K quality were printed using SG100 resin (eSUN, Shenzhen, China).

### Filling C-shaped replicas

The replicas were obturated with either E. TCS (Maruchi, Wonju, Korea) or E. MTA (Maruchi, Wonju, Korea) and the following techniques:

1. E. MTA/E. TCS + single-cone technique (SC) [15]: The sealer was dispensed directly into the middle third of the mesial and distal C-shaped canal according to the manufacturer’s instruction from a premixed syringe via a disposable canal tip. Sealer injection continued until it was observed in the root canal orifice. Two selected master gutta-percha cones (0.04/#35) (Sure Dent, Gyeonggi-do, South Korea) were placed slowly in the mesiobuccal and distobuccal parts of the C-shaped canal with 2–3 times pumping motion. The extended ends of cones were seared off at the canal orifice level, and no additional cones were used.
2. E. MTA/E. TCS + single-cone technique with ultrasonic activation (UA) [3]: After placing the sealer into the canal, ultrasonic vibration was applied to a cotton plier that held the gutta-percha cone 20 mm from its tip using an ultrasonic tip (E7, Eighteeth, Jiangsu, China). Then, the cone slowly reached the working length during continuous ultrasonic activation. The ultrasonic application time during gutta-percha cone placement was 2–3 s, and the excess cone was cut at the orifice level.
3. E. MTA/E. TCS + cold hydraulic compaction [17]: After injecting sealer into the canal, one master gutta-percha cone (0.04/#40) (Sure Dent, Gyeonggi-do, South Korea) was coated with sealer and inserted into the working length. A root canal spreader was passively inserted (without lateral compaction force) into the main canals and isthmus area to approximately 2 mm from the working length. Accessory cones (0.02/#25) (Sure Dent, Gyeonggi-do, South Korea) were inserted into the spaces. The obturation procedures were repeated until the canals were filled. The gutta-percha cones were seared off at the orifice.

An endodontist conducted root canal preparation and obturation. The time required to fill each sample was recorded from the moment the master cone was inserted into the canal until the excess cone was cut at the orifice level. Apical extrusion of gutta-percha was noted using a yes or no scheme (19). Afterward, all filled samples were incubated at 37° degrees in 100% humidity for one week to ensure a complete sealer setting.

### Measuring sealer and void percentage

**Fig. 1:**
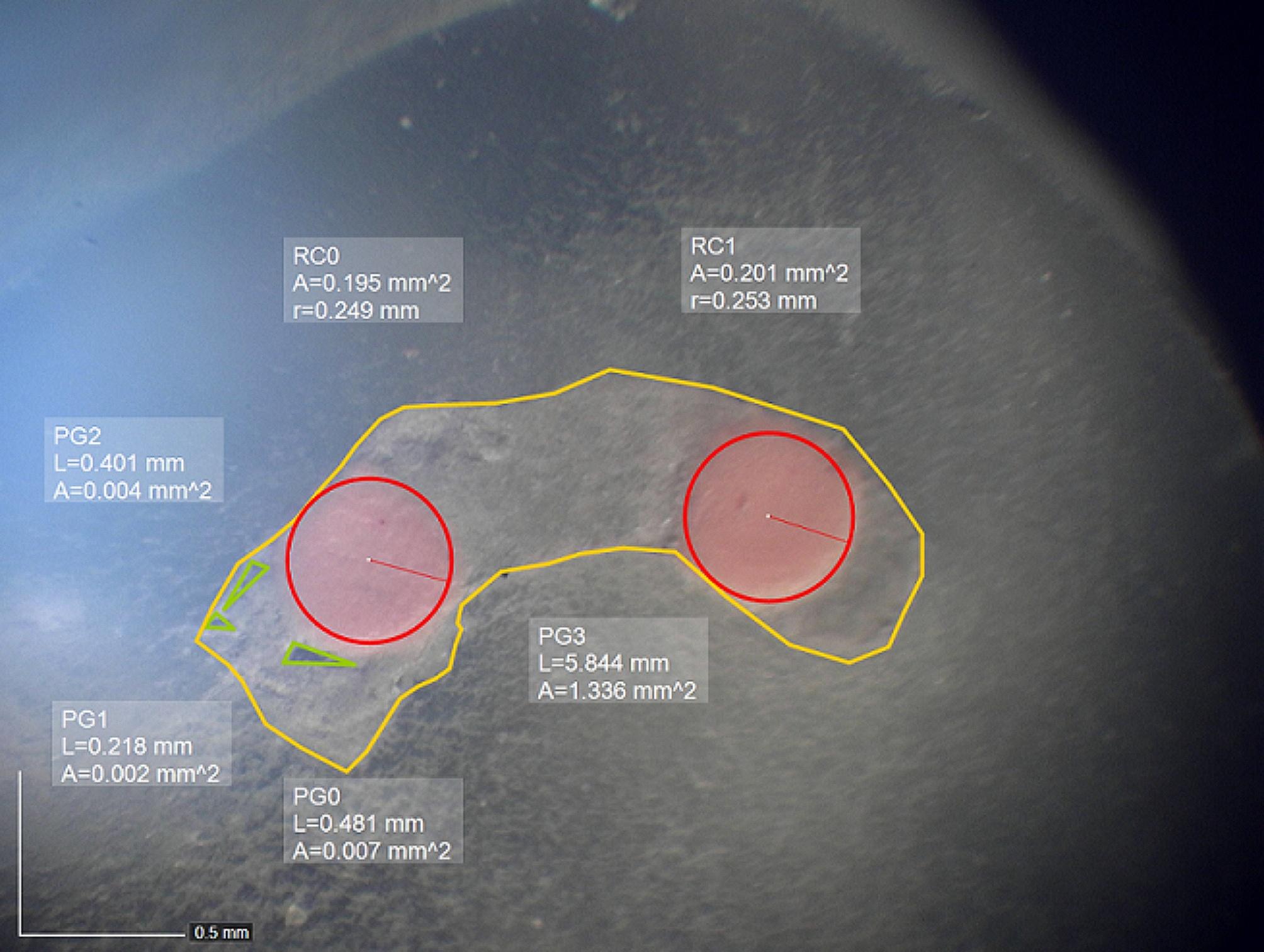
Measurements of the gutta-percha (red frame), sealer (yellow frame), and void (green frame) percentages in the middle third of a replica obturated with EndoSeal MTA without ultrasonic activation in 100x magnification

All samples were sectioned using a 0.3 mm disc of a CNC cutting machine (Delta Electronics, Inc., Taoyuan, Taiwan) with copious water irrigation at 2, 5, and 7 mm from the apex. Images of the thirds were acquired using a Bluelight stereomicroscope (Blue Light Industry USA Inc., CA, USA) with x32 magnification. The images were analyzed in DinoCapture 2.0 software. Areas comprising sealer and voids were expressed as percentages of the total area [19] (Fig. 1).

### Statistical analysis

The SPSS software 23.0 (IBM Inc., NY, USA) was used for data analysis. One-way ANOVA analysis and LSD post-hoc test were used to compare the treatment time between the groups. Sealer extrusion differences between the groups were compared using the Chi-Square test. The significance level was set at 0.05.

## Results

According to Table 1, void and sealer percentages differed significantly between the groups in all thirds (*p* < 0.001). In the apical third, E. TCS with or without ultrasonic activation and E. MTA with ultrasonic activation showed the lowest void percentages among groups (1.51 ± 0.68%, 1.04 ± 0.22%, and 1.52 ± 0.80%, respectively; *p* < 0.05). These groups also showed the highest sealer percentages in the apical third (*p* < 0.05).

**Table 1:** The mean ± standard deviation of sealer and void as the percentage of the root canal sections area in apical, middle, and coronal thirds for each obturation technique

Void or sealer	Section	Sealer and activation mode						*P* value
		E. MTA-SC	E. MTA-UA	E. MTA-CHH	E. TCS-SC	E. TCS - UA	E. TCS -CHC	
Void	Apical	2.06 ± 0.70 A	1.52 ± 0.80AB	4.01 ± 0.41 C	1.51 ± 0.68B	1.04 ± 0.22B	3.10 ± 0.60D	**< 0.001**
	Middle	3.33 ± 0.62 A	2.58 ± 0.77B	3.10 ± 0.82AB	2.55 ± 0.65BC	1.70 ± 0.43D	2.18 ± 0.56CD	**< 0.001**
	Coronal	4.15 ± 0.76 A	3.56 ± 0.46B	2.00 ± 0.54 C	3.39 ± 0.45B	2.28 ± 0.44 C	1.09 ± 0.41E	**< 0.001**
Sealer	Apical	58.52 ± 0.61 A	59.26 ± 1.25AB	45.17 ± 1.28 C	59.38 ± 1.09AB	59.82 ± 0.66B	36.71 ± 1.09D	**< 0.001**
	Middle	67.63 ± 2.36 A	67.74 ± 1.48 A	56.24 ± 1.48 C	68.27 ± 1.00AB	69.23 ± 0.73B	42.40 ± 1.82D	**< 0.001**
	Coronal	73.78 ± 1.29 A	74.33 ± 0.79AB	57.88 ± 1.11 C	74.46 ± 1.09AB	75.46 ± 0.83D	40.13 ± 1.25E	**< 0.001**

SC: Single-cone; US: Ultrasonic activation; CHC: Cold hydraulic compaction

*Values less than 0.05 represent a significant difference between the groups using one-way ANOVA test (*p* < 0.05)

In each row, different uppercase represents a significant difference according to LSD post-hoc test (*p* < 0.05)

E., EndoSeal; UA, Ultrasonic activation; CHC, Cold hydraulic compaction

In the middle third, samples obturated by E. TCS with ultrasonic activation and CHC showed the lowest void percentages among the groups (1.70 ± 0.43% and 2.18 ± 0.56%, respectively; *p* < 0.05). In this third, E. TCS with or without ultrasonic activation and E. MTA with ultrasonic activation had the highest sealer percentages (59.82 ± 0.66%, 59.38 ± 1.09%, and 59.26 ± 1.25%, respectively; *p* < 0.05).

In the coronal third, E. TCS with cold hydraulic compaction showed the lowest void percentage among the groups (1.09 ± 0.41%; *p* < 0.05). However, the E. TCS with ultrasonic activation had the highest sealer percentage among the groups (75.46 ± 0.83%; *p* < 0.05).

Obturation duration for each group is provided in Table 2. According to the one-way ANOVA test result, treatment time was significantly different among the groups (*p* < 0.001). The results of the LSD post-hoc test showed that obturation with E. TCS with or without ultrasonic activation needed significantly lower obturation time among groups (72.50 ± 6.34 s and 82.50 ± 11.84 s, respectively; *p* < 0.05).

**Table 2:** The mean ± standard deviation of treatment duration (seconds) for each obturation technique

Sealer and activation mode	Time (Second)	*P* value
E. MTA-SC	87.50 ± 13.79 A	< 0.001^a)^
E. MTA-UA	107.50 ± 13.79B	
E. MTA-CHC	159.50 ± 18.47 C	
E. TCS-SC	72.50 ± 6.34D	
E. TCS-UA	82.50 ± 11.84AD	
E. TCS-CHC	159.50 ± 18.47E	

SC: Single-cone; US: Ultrasonic activation; CHC: Cold hydraulic compaction

a) Values less than 0.05 represent a significant difference between the groups using one-way ANOVA test (*p* < 0.05)

Different uppercase represents a significant difference according to LSD post-hoc test (*p* < 0.05)

E., EndoSeal; UA, Ultrasonic activation; CHC, Cold hydraulic compaction

The number of teeth with evidence of sealer extrusion in each group is provided in Table 3. The Chi-Square test revealed that the sealer extrusion significantly differed among the groups (*p* = 0.019). E. TCS and E. MTA with ultrasonic activation showed higher sealer extrusion than other groups (60%).

**Table 3:** Comparison between the number of teeth with sealer extrusion among the groups

Sealer and activation mode	Yes	No	Total	*P* value
E. MTA-SC	1	9	10	0.019^a)^
E. MTA-UA	6	4	10	
E. MTA-CHC	4	6	10	
E. TCS-SC	5	5	10	
E. TCS-UA	6	4	10	
E. TCS-CHC	4	6	10	

SC: Single-cone; US: Ultrasonic activation; CHC: Cold hydraulic compaction

^a)^Value less than 0.05 represent a significant difference between the number of teeth with sealer extrusion among the groups based on the Chi-Square test

E., EndoSeal; UA, Ultrasonic activation; CHC, Cold hydraulic compaction

## Discussion

The present study compared the obturation quality of 3D-printed C-shaped canal replicas with C1 configuration filled with two bioceramic sealers and single cone technique with or without ultrasonic activation and CHC method. The findings highlighted a significant difference in the void and sealer proportion across the groups. Specifically, utilizing E. TCS and ultrasonic activation resulted in the least amount of voids in the apical and middle thirds of the root canal. In the coronal third, the E. TCS sealer combined with the CHC technique displayed the lowest percentages of voids among all groups, followed by the obturating with E. TCS and ultrasonic activation, and E. MTA and the CHC technique.

We utilized 3D-printed replicas of C-shaped canals to standardize the samples in our experiment. Previous studies have explored various C-shaped canal obturation techniques with resin sealers using printed resin replicas [19, 20]. However, research on obturating C-shaped canals with bioceramic sealers is scarce [17]. Given the bioceramic sealers’ favorable properties, easy application, and capability to reach anatomically challenging areas, they emerge as potential filling material in the root canal treatment of teeth with complex anatomies [4]. To the best of our knowledge, this research is the pioneering study to compare different obturation techniques employing bioceramic sealers in C-shaped canal printed replicas.

Some studies prepared the C-shape canals up to F3 and F2 files [19]. In our study, similar to Zhao et al. [21], preparation was limited to the #30 file of 4% due to the thin apical area of the C-shaped canal.

The void percentage assessment has been used as a common method in in vitro study to evaluate the quality of root canal fillings [20, 22, 23]. Bacteria and their byproducts may remain at a site in void areas, affecting the treatment’s success [23].

Root canal filling quality is frequently evaluated with Micro-CT due to its ability to precisely detect void location and volume. Yet, since most sealers are opaque, micro-CT might overlook smaller voids within the root-filling materials [15]. Recent research suggests that micro-CT could be less sensitive than sectioning methods in detecting voids [15]. Therefore, we employed the sectioning method and a digital microscope in our study to enhance the accuracy of void detection.

We employed the single cone technique for root canal filling using bioceramic sealers due to its heightened bond strength and decreased microleakage [24]. Furthermore, studies have indicated that this technique results in comparable void volume and percentage with other obturation methods with various sealers [25–28]. Keles et al. [25] found a similar void percentage following obturating the band-shaped isthmuses by warm vertical compaction (13.11%) and single-cone technique (15.60%) with AH Plus sealer. Maddalone et al. [26] also found a similar void percentage following obturating extracted teeth with the GuttaFlow bioseal group and BioRoot RCS with the single-cone technique. Alim et al. [27] found that obturating severely curved canals with lateral condensation technique, single-cone technique, continuous-wave obturation technique, and core carrier technique with the AH Plus sealer led to comparable filling material percentage. According to Kim et al. [28], obturating the palatal canal of extracted maxillary molars with Endoseal MTA or AH Plus Jet using the single gutta-percha cone technique led to comparable void percentage between groups.

Samples obturated with CHC techniques showed a significantly higher void percentage among groups in the apical third. Wisawantin et al. [17] reported similar findings with obturating natural C-shaped canals with bioceramic sealers and CHC technique compared with epoxy-resin sealers. They also reported spreader tracts in samples obturated with this technique. Therefore, the compaction force from a root canal spreader might be insufficient to push the sealer into the isthmus or apical narrow areas. According to our findings, although the CHC technique showed a comparable void percentage in the middle and coronal thirds to other methods, it was significantly more time-consuming.

In the apical third, E. TCS ultrasonic activation resulted in fewer voids than E. TCS without activation. However, the difference was not statistically significant. In the middle third, specimens obturated with E. TCS activation led to the least voids among all groups. This technique was simple and relatively less time-consuming than other methods. Numerous studies highlight the advantages of ultrasonic activation across various sealers, especially bioceramic sealers, in enhancing root canal filling quality [16, 29–31]. Generated heat during activation decreases sealer viscosity and, coupled with the acoustic streaming energy, ensures the sealer disperses and adheres to the canal walls [11]. Ko et al. [32] also reported that ultrasonic activation of the E. TCS sealer in ribbon-shaped canals significantly reduced voids compared to when the sealer was not activated. Damade et al. [33], noted that oval-shaped canals filled with bioceramic sealer using the single cone technique had more voids than those filled using the warm vertical compression technique. Nevertheless, the void presence in both techniques was comparable after ultrasonic activation. Kim et al. [15] found that after ultrasonically activating the E. MTA sealer, the void filling in oval-shaped canals was on par with the AH Plus sealer used with the warm vertical compaction method.

An endodontic sealer’s flow is an influential factor in obturating anatomical irregularities, such as isthmus and lateral canals [34]. The E. TCS sealer has a lower viscosity than the E. MTA sealer (approximately 19 mm compared to 17 mm) [8, 35]. In our study, samples obturated with E. TCS had a lower void proportion than E. MTA in all thirds. However, following ultrasonic activation of E. MTA sealer, comparable results were achieved to E. TCS sealer.

Sealers with lower viscosity are more prone to extrusion from the apical foramen [8]. Our findings indicated a higher sealer extrusion rate in the E. TCS group compared to E. MTA. Ultrasonic activation further amplified this tendency. In the clinical trial of Kim et al. [36], the sealer extrusion rate after root canal treatment using E. TCS sealer was approximately 37%, aligning with our observations. However, Fonseca et al. [37] reported that even though teeth obturated with bioceramic sealer had more apical extrusion than those with resin sealer, post-treatment pain levels were similar. Chybowski et al. [38] found a 47.5% apical extrusion rate with the EndoSequence bioceramic sealer, but this wasn’t linked to treatment outcomes at a 30-month follow-up. Still, caution is advised when using these sealers near the mandibular nerve canal, especially for C-shaped canals commonly found in mandibular second molars [39].

Our study faced some limitations. Its laboratory setting might not perfectly replicate the oral cavity’s temperature conditions, potentially affecting void amounts. To address this, we incubated the teeth at 37 degrees for a week. Additionally, the pressure from periodontal tissue might counteract the apical extrusion of the sealer [40]. We also focused solely on the C1 anatomy of C-shaped canals and used two types of sealers. Future in vitro studies using printed resin samples could expand on our findings by exploring other C-shaped canal anatomies and different bioceramic sealers. Clinical studies investigating various methods and sealers and their impact on the success of endodontic treatment of C-shaped canals would also be valuable.

Within the limitations of this study, it was concluded that using the E. TCS sealer with ultrasonic activation yielded the best obturation quality and the lowest amount of void in 3D-printed C-shaped canals. Moreover, this technique was less time-consuming than other obturation methods. Sealer extrusion incidence associated with this technique was higher than other techniques, and care must be taken when obturating the canals with high flow and ultrasonic activation near the mandibular nerve canal.

## Data Availability

The datasets used and/or analyzed during the current study available from the corresponding author on reasonable request.
